# The Potential Use of 11C-Choline Positron Emission Tomography/Computed Tomography to Monitor the Treatment Effects of Radium-223 in a Patient with Prostate Cancer

**DOI:** 10.7759/cureus.2948

**Published:** 2018-07-09

**Authors:** Kazuhiro Kitajima, Shingo Yamamoto, Norihiko Kamikonya, Yukako Nakanishi, Yusuke Yamada, Takahiko Hashimoto, Toru Suzuki, Shuken Go, Akihiro Kanematsu, Michio Nojima, Koichiro Yamakado

**Affiliations:** 1 Radiology, Hyogo College of Medicine, Nishinomiya, JPN; 2 Urology, Hyogo College of Medicine, Nishinomiya, JPN; 3 Urology, Hyogo College of Medicine, Nisnomiya, JPN

**Keywords:** prostate cancer, ra-223, pet, choline

## Abstract

We report here about a 59-year-old man with bone metastatic castration-resistant prostate cancer and biochemical progression, who underwent radium-223 (Ra-223) therapy, following previous treatment failure. Treatment response of osseous metastases was assessed with three ^11^C-choline positron emission tomography/computed tomography (PET/CT) scans at baseline, after three cycles for early monitoring, as well as after six cycles of radium-223 therapy. Pretreatment ^11^C-choline PET/CT showed multiple areas of increased focal activity in multiple cervical, thoracic, and lumbar vertebrae as well as in both ribs, right ileum, and left ischium. Second ^11^C-choline PET/CT after three cycles showed increasing tumor activity in the existing lesions and the new uptake spots of thoracic spine, both ribs and left ileum. Third ^11^C-choline PET/CT at the end of the therapy showed further progression with new lesions of thoratic spine, sacrum, right rib, and right ileum. In this case, ^11^C-choline PET/CT after three cycles for early monitoring could predict the therapeutic response to Ra-223.

## Introduction

Radium-223 (Ra-223) dichloride, an alpha particle emitting therapeutic radiopharmaceutical, is an approved therapy for the treatment of metastatic castration-resistant prostate cancer (CRPC) with symptomatic osseous metastases but no visceral metastases, and provides improved survival [1-2]. Currently, there is clinically no diagnostic test known to reliably measure or predict the therapeutic response to Ra-223.

Although bone scintigraphy is a standard method of choice to assess the bony metastasis of prostate cancer and continues to be used in terms of cost, availability, and execution in clinical practice [3], it is not a perfect tool in terms of both sensitivity and specificity. In this context, positron emission tomography/computed tomography (PET/CT) using new tracers such as fluoride [4], choline [5-7], and prostate specific membrane antigen (PSMA) [8] has emerged as a useful method.

We report here a case in which three 11C-choline PET/CT scans (before, during, after Ra-223) were used to diagnose skeletal tumor metabolic activity and to assess and predict the treatment response of Ra-223 in a patient with CRPC.

## Case presentation

A 59-year-old man had undergone prostate needle biopsy after a high prostate specific antigen (PSA) level (218.5 ng/mL) observed at the age of 54 years and he was diagnosed with adenocarcinoma of the prostate (Gleason score 4＋5). He underwent pelvic magnetic resonance imaging (MRI) and bone scintigraphy at our hospital. The MRI showed the mass in the right peripheral zone as a low signal intensity on the T2-weighted image and as an abnormal signal intensity on the diffusion-weighted image, reflecting prostate cancer. Bone metastases of the right rib and L2 vertebra were clarified. Therefore, clinical stage was T2aN0M1. He was treated with radiation therapy and androgen deprivation therapy (ADT) including bicalutamide and goserelin and his PSA dropped to 0.053 ng/mL. At the age of 56 years, his PSA was found to be elevated (1.15) and ADT with flutamide, estramustine phosphate, enzalutamide, and abiraterone was restarted. At the age of 58 years, the disease became refractory to hormonal treatment (PSA recurrence: 24.9 ng/mL), and the patient started chemotherapy with docetaxel for six cycles. However, both symptomatic and biochemical progression (PSA: 33.7 ng/mL) appeared. We confirmed multiple bone metastases without lymph node metastases or visceral metastases by carrying out baseline 11C-choline PET/CT and started Ra-223. He completed all the six cycles without any interruption and with no adverse events. Before each treatment, laboratory evaluation was performed to assess hematological parameters as well as PSA. After treatment cycles 3 and 6, 11C-choline PET/CT imaging studies were performed to evaluate and predict treatment response of Ra-223 on imaging.

**Figure 1:**
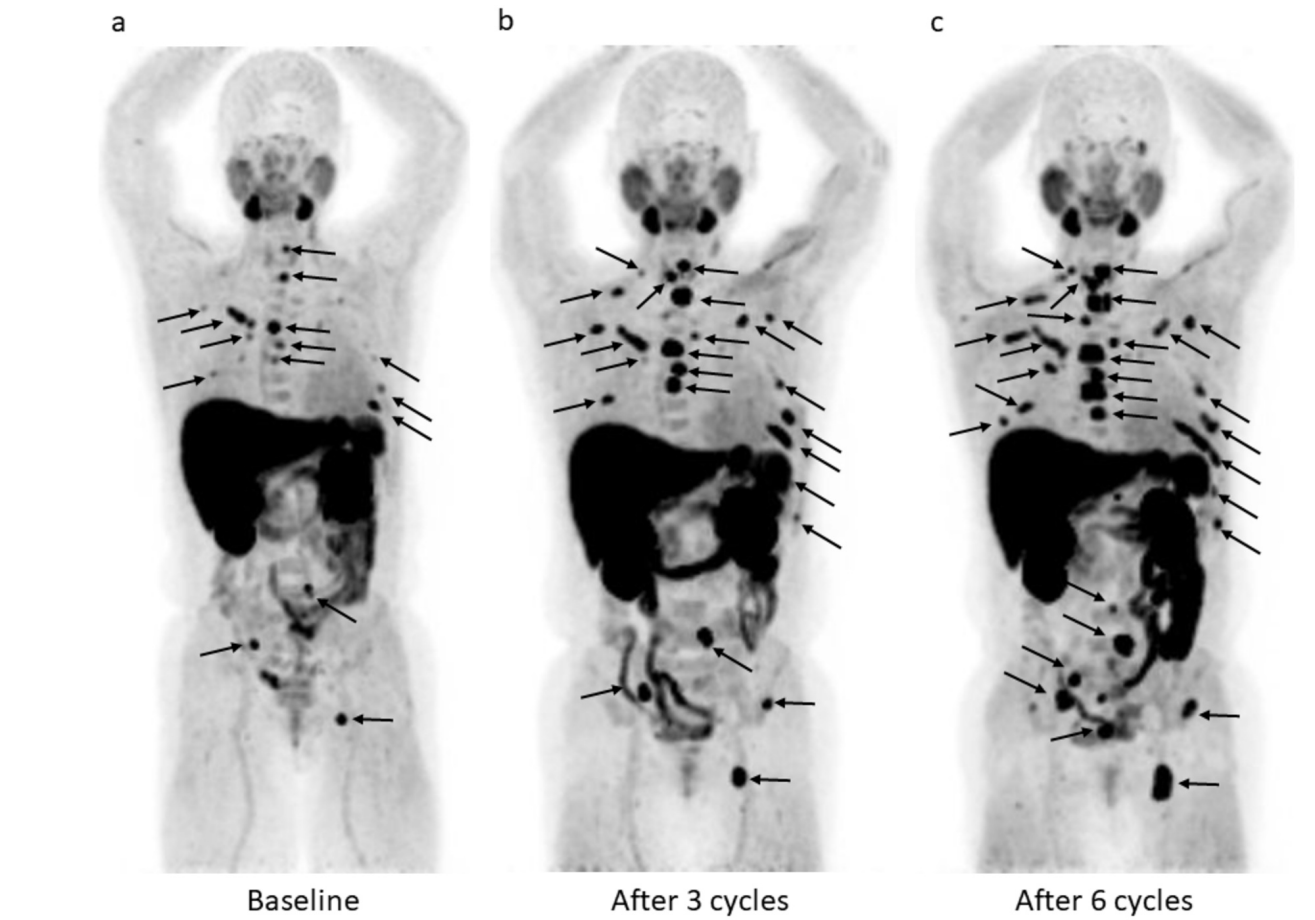
A 59-year-old man with bone metastatic castration-resistant prostate cancer. Maximum intensity projection (MIP) images in the coronal projection of sequential ^11^C-choline PET/CT scans carried out at (a) baseline, (b) after three cycles, and (c) after six cycles of Ra-223 therapy. (a) Pretreatment MIP image showing multiple areas of increased focal activity in the spine, ribs, and the pelvic bone (arrows). (b) MIP image after three cycles showing increasing tumor activity in the existing lesions and the new uptake spots of spine, ribs, and pelvic bone (arrows). (c) Post-treatment MIP image showing further progression with new lesions (spine, ribs, and pelvic bone) (arrows).

**Figure 2:**
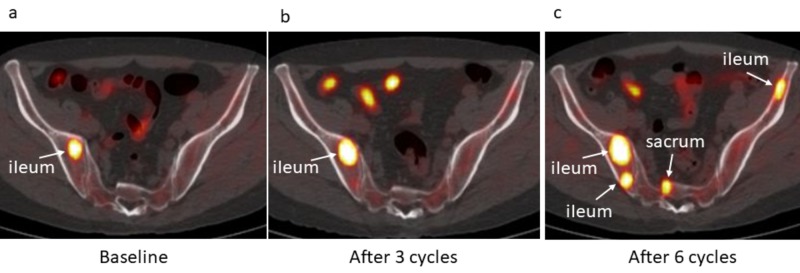
Selected axial 11C-choline PET/CT plane at (a) baseline, after (b) three and (c) six cycles. (a) A lesion in right ileum showed maximum standardized uptake value (SUVmax) of 8.1 on pretreatment ^11^C-choline PET/CT (arrow), reflecting bone metastasis. (b) The SUVmax of right iliac lesion increased to 14.3 on ^11^C-choline PET/CT after three cycles (arrow), reflecting the progression of bone metastasis. (c) The SUVmax of right iliac lesion kept high level (14.4) on ^11^C-choline PET/CT after six cycles. Several new uptakes appeared on the right ileum (SUVmax, 7.2), left ileum (SUVmax,5.0) and sacrum (SUVmax, 4.7), respectively (arrows), reflecting new bone metastases.

**Figure 3:**
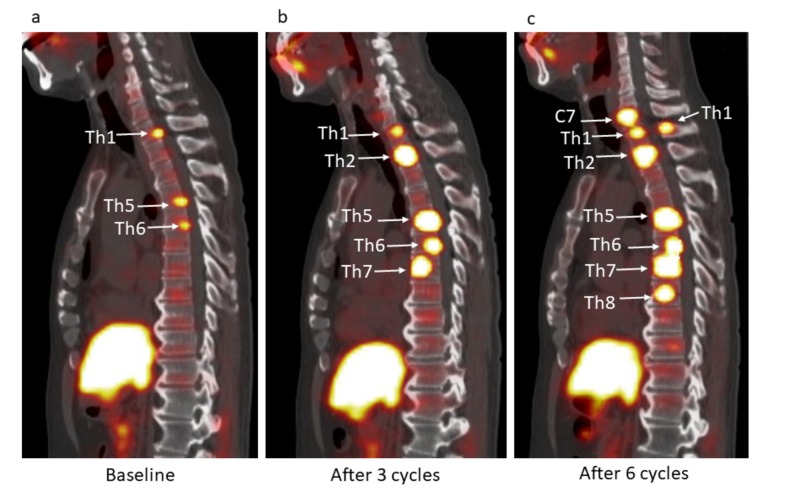
Selected sagittal 11C-choline PET/CT plane at (a) baseline, after (b) three and (c) six cycles. (a) Three hot spots were observed in Th1 vertebra (SUVmax, 4.9), Th5 vertebra (SUVmax, 4.7), and Th6 vertebra (SUVmax, 4.3) on pretreatment ^11^C-choline PET/CT (arrows), reflecting bone metastases. (b) Five hot spots were observed in Th1 vertebra (SUVmax, 4.9), Th2 vertebra (SUVmax, 15.0), Th5 vertebra (SUVmax, 18.1), Th6 vertebra (SUVmax, 12.8), and Th7 vertebra (SUVmax, 13.2) on ^11^C-choline PET/CT after three cycles (arrows), reflecting the progression of bone metastases. (c) Eight hot spots were observed in C7 vertebra (SUVmax, 11.1), Th1 vertebra (SUVmax, 5.7) Th1 spinous process (SUVmax, 6.2), Th2 vertebra (SUVmax, 14.4), Th5 vertebra (SUVmax, 16.2), Th6 vertebra (SUVmax, 12.0), Th7 vertebra (SUVmax, 12.6), and Th8 vertebra (SUVmax, 12.2) on ^11^C-choline PET/CT after six cycles (arrows), reflecting further progression of bone metastases.

Baseline 11C-choline PET/CT showed multiple areas of increased focal activity in multiple cervical, thoracic and lumbar vertebrae as well as in both ribs, right ileum, and left ischium (Figures 1a-3a). Second 11C-choline PET/CT after three cycles showed the increasing tumor activity in the existing lesions and the new uptake spots of thoracic spine, both ribs, and left ileum (Figures 1b-3b). Third 11C-choline PET/CT after six cycles showed the increasing tumor activity in the existing lesions and the new uptake spots of thoracic spine, sacrum, right rib, and right ileum (Figures 1c-3c).

The patient’s serum alkaline phosphatase (ALP) level was 226 U/L at baseline, 191 U/L after one cycle, 208 U/L after two cycles, 235 U/L after three cycles, 206 U/L after four cycles, 200 U /L after five cycles, and 262 U/L after six cycles, respectively. His serum PSA level was 33.7 ng/mL at baseline, 54.1 ng/mL after one cycle, 79.4 ng/mL after two cycles, 85.6 ng/mL after three cycles, 120 ng/mL after four cycles, 175 ng/mL after five cycles, and 267 ng/mL after six cycles, respectively. During Ra-223 therapy, his serum ALP did not dramatically change with minor increase and decrease and PSA gradually increased. After the completion of Ra-223 therapy, he started a new chemotherapy with cabazitaxel.

## Discussion

Bone metastases are a primary factor contributing to CRPC morbidity and mortality. One problem in treating CRPC is the lack of reliable methods for assessing skeletal treatment response in real time. Indeed, bone lesions are generally considered nonmeasurable by the Response Evaluation Criteria in Solid Tumors (RECIST) [9]. This real difficulty in assessing skeletal tumor response in CRPC not only hampers clinical practice, but also impedes drug development. An imaging method capable of assessing tumor response in a timely fashion may not only speed up the discovery of new treatments for CRPC, but also provide potentially predictive information that could help tailor therapy for individual patients. In the Ra-223 phase 3 trial, a total ALP response (≥30%decline or >50 % reduction from baseline) was observed more frequently in the Ra-223 treated arm versus placebo [1], while there is evidence suggesting that bone ALP is a tumor response marker in CRPC [10].

Several groups have demonstrated the potential use of choline PET/CT during the Ra-223 treatment for assessing and predicting the therapy effects in the form of case reports [5-7]. Only two papers [5, 7] have scanned two 18F-choline PET/CT before and after three cycles of Ra-223 and demonstrated that 18F-choline PET/CT after three cycles could assess and predict treatment response of Ra-223 in a total of five patients with CRPC similar to our series. Miyazaki et al. [5] demonstrated two cases, one case with near-total disappearance of abnormal skeletal activity and another case with heterogeneous tumor response. García Vicente et al. [7] compared 18F-choline PET/CT and bone scintigraphy in three cases and demonstrated that both modalities are in agreement with the biochemical response, although differences in the disease expression (concordant and discordant patterns) were found because of the different radiotracer biodistribution and molecular information derived from them.

The 18F-NaF bone imaging is gaining widespread use in detecting osteoblastic metastases and 18F-NaF PET/CT has been reported to be superior to 18F-choline PET/CT for detecting osteoblastic metastases of prostate cancer [11]. Its application in therapy assessment is, however, still a matter of debate, mainly due to flare phenomenon [12]. Another study evaluated the capability of 18F-NaF PET/CT to determine the response to 223Rain 10 mCRPC patients. Tumor quantification was performed in five different regions at baseline, after the first Ra-223 cycle (interim PET) and after the sixth Ra-223 cycle. While changes in 18F-NaF PET/CT between the baseline and the end-of-treatment image were able to indicate response to therapy (albeit in a univariate model), the interim 18F-NaF PET/CT imaging did not prove useful [13].

It is still not clear as to which tracer will be appropriate in evaluating response to Ra-223, whether 18F-NaF or specific tracers. There have been no reports comparing 18F-NaF and choline PET/CT to monitor the treatment effects of Ra-223. In Japan, choline, 18F-NaF, and 68Ga-PSMA were recently introduced for clinical use in Western countries, but they are not yet freely available and covered by health insurance in Japan.

## Conclusions

Our case report illustrates the potential use of ^11^C-choline PET/CT to monitor the effects of Ra-223 on skeletal tumor metabolic activity. Clinical outcome studies in a sufficient number of patients will be needed to ascertain the therapeutic predictive value of 11C-choline PET/CT.
